# A Data Engineering Framework for Ethereum Beacon Chain Rewards: From Data Collection to Decentralization Metrics

**DOI:** 10.1038/s41597-025-04623-7

**Published:** 2025-03-28

**Authors:** Tao Yan, Shengnan Li, Benjamin Kraner, Luyao Zhang, Claudio J. Tessone

**Affiliations:** 1 Blockchain & Distributed Ledger Technologies Group at Department of Informatics and UZH Blockchain Center, University of Zurich, 8050 Zurich, Switzerland; 2 Data Science Research Center and Social Science Division, Duke Kunshan University, Suzhou, 215316 China

**Keywords:** Economics, Electrical and electronic engineering, Computational science, Technology, Industry

## Abstract

Ethereum, one of the leading smart contract blockchain platforms, currently operates on a Proof-of-Stake (PoS) consensus mechanism designed to secure the network while incentivizing desired validator behaviors. Despite blockchain technology’s promise of decentralization, limitations and gaps in decentralization persist, posing challenges for analysis and optimization. This study introduces a comprehensive dataset of validator rewards from the Ethereum Beacon chain, categorized into attestation, proposer, and sync committee rewards. By providing granular, transparent, and auditable records of validator activities, the dataset addresses the fragmentation of raw blockchain data and enables robust evaluations of PoS incentive structures. Researchers can leverage this dataset to assess enforceable rules, verify protocol compliance, and analyze long-term validator behavior. In addition, we apply decentralization metrics such as the Shannon entropy, Gini Index, Nakamoto Coefficient, and Herfindahl-Hirschman Index (HHI) to showcase the dataset’s utility in studying decentralization trends. Publicly available on Harvard Dataverse and accompanied by open-source analytical tools on GitHub, this dataset facilitates future research aimed at enhancing blockchain systems’ decentralization, security, and efficiency.

## Background & Summary

Blockchain technology is designed to catalyze a shift towards a decentralized and equitable digital ecosystem. However, previous studies have revealed significant centralization tendencies in Ethereum during its Proof-of-Work (PoW) phase, both in terms of transaction network patterns^1,2^ and wealth distribution characteristics^3,4^. The introduction of Proof-of-Stake (PoS) Ethereum signifies a transformative development in the realm of blockchain technology, marking a departure from the established Proof-of-Work to the Proof-of-Stake consensus mechanism^5–7^. This transition, which took place on September 15, 2022, not only aims to mitigate the environmental and scalability challenges inherent in PoW but also ushers in a novel approach to reward distribution that prioritizes staking Ether over computational exertion. Previous studies have underscored significant variations in reward distribution among different blockchain networks, such as Tezos, Polkadot, Cardano, Casper^8^ and Bitcoin^9–11^, sparking debates over the potential concentration of wealth and authority^12–16^. Against this backdrop, Ethereum’s PoS iteration offers an invaluable opportunity to investigate whether this paradigm shift could lead to a more equitable allocation of rewards, challenging the centralization trends noted in PoW frameworks.

Ethereum is one of the few blockchains to have transitioned from PoW to PoS. This transition introduces significant changes in data accessibility. Unlike PoW, where reward data is readily accessible on the execution layer, PoS reward data resides on the Beacon chain, which significantly increases the complexity of data collection and analysis. There is a noticeable scarcity of detailed, accessible data on reward distribution within the PoS stage of Ethereum. Many existing studies rely on data obtained directly from APIs or third-party sources, which often raises concerns about the completeness and accuracy of the dataset. At the time of writing, few third-party blockchain data platforms offer comprehensive Beacon chain reward data. Notably, mainstream platforms like Dune, Nansen, and Google BigQuery do not provide such data. Furthermore, few studies have clearly addressed the technical challenges and requirements associated with collecting and parsing raw data directly from the Ethereum Beacon chain. In parallel, while some research has provided theoretical insights into the decentralization of PoS systems^17,18^, and examined the centralization of crypto-asset holdings in many blockchains, both PoW-based and PoS-based systems, particularly in terms of wallet and asset distribution^19^, comparative studies evaluating similar dynamics within Ethereum’s evolving PoS ecosystem^20^ remain notably limited. This lack of methodologies for Ethereum further underscores the need for a systematic approach to data collection and analysis on blockchain decentralization. Our study seeks to bridge these gaps by formulating a comprehensive methodology for accruing reward data from the Ethereum Beacon chain, with the goal of providing decentralization metrics in this emergent ecosystem. By implementing Ethereum Erigon and Teku nodes to harvest data from the Beacon chain and employing a variety of metrics to scrutinize the decentralization of reward allocation, our research enables a detailed examination of decentralization metrics in Ethereum’s PoS ecosystem. Our paper has three main contributions, which are as follows:

1. We develop a systematic node-based methodology to collect Ethereum’s PoS reward data, thereby overcoming the incompleteness of third-party datasets. This approach enables a more accurate and comprehensive decentralization analysis in Ethereum’s PoS ecosystem where empirical studies remain limited. This dataset, significant for its relevance and scope, has been made publicly accessible to facilitate and encourage further scholarly research.
2. Our data engineering framework lies in its capability to capture validator rewards at their fundamental granularity levels on the Ethereum Beacon chain. While our published dataset provides daily aggregated metrics for practical storage and usage considerations, the framework itself supports the extraction of rewards at their finest intervals: proposer and sync committee rewards at slot level (every 12 seconds) and attestation rewards at epoch level (every 6.4 minutes). This granular data collection capability offers researchers unprecedented visibility into Ethereum’s reward mechanisms, validator performance variations, and network participation dynamics.
3. Our dataset lends itself to a variety of analytical applications, encompassing not only time-series analysis but also an exploration of inter-layer blockchain decentralization. Furthermore, it provides a foundation for a comparative analysis of reward distribution between PoS and PoW blockchain architectures, offering critical insights into the evolving landscape of blockchain technology.

To the best of our knowledge, this is the first study to systematically describe how to collect and structure the Ethereum Beacon chain reward data, as well as to scrutinize the reward decentralization at the validator index level. While previous studies have delved into the decentralization of wealth in blockchain systems like Cardano, Bitcoin^21^, and the PoW Ethereum^22^, our focus is on the PoS Ethereum, thus providing fresh insights into its reward dynamics.

The organization of this manuscript is outlined as follows: the Method section elucidates our research methodology, encompassing the deployment of archive nodes, decomposition of the Beacon rewards, and the application of various inequality metrics. The Data Records section systematically presents the data record. In the Technical Validation section, we undertake a technical validation of our methodology, contrasting it with additional data sources for robustness. Section Usage Notes delves into the potential applications of our datasets, highlighting their versatility and scope. Finally, the Future Research section details the accessibility of our open-source code, underlining our commitment to transparency and collaborative research.

## Methods

**Fig. 1:**
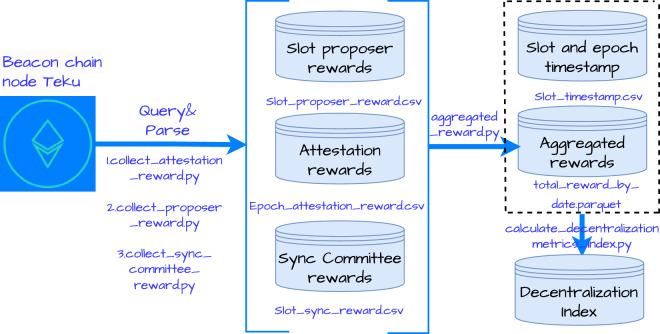
The data engineering workflow for the Ethereum Beacon chain rewards.

In this section, we present the data engineering workflow, exemplified in Fig. 1.

### Deployment of archive node for blockchain data collection

To acquire reward data for the PoS Ethereum, we deployed consensus and execution nodes, utilizing the Teku at https://github.com/Consensys/teku and Erigon at https://github.com/erigontech/erigon clients on the Linux server. Both clients are archive nodes that can maintain the entire historical state of the blockchain, especially the account balances at every block which the full blockchain nodes cannot offer. We show the experiment setting in Table 1. Subsequently, after synchronizing the block data, we employed the *Web3.py* Python library and the API of the Teku node to collect reward data. The rewards are categorized into three main types: proposer reward, attestation reward, and sync committee reward. Notably, the attestation reward is updated per epoch. Each epoch can encompass hundreds of thousands, or even millions, of validators, leading to a substantial volume of data. We collected data for one year after the Ethereum PoS transition, resulting in a total dataset size of 1.7 terabytes, with attestation reward data constituting 1.6 terabytes. All the reward data obtained are in Gwei, which is equivalent to 10^−9^ Ether. We convert the rewards from Gwei to Ether in our analysis.

**Table 1:** Experiment setting.

Component	Specification
CPU	128 cores
RAM	512 GB
Storage	10 TB SSD
Operating System	Debian 5.10.162-1
Erigon client version	erigon v2.60.10 linux amd64
Teku client version	teku 24.6.1
Web3.py version	6.14.0

### Decomposition of validator and beacon rewards by sources

The *PoS Ethereum* incorporates a multifaceted reward system designed to incentivize validators across various dimensions of system participation and security. Notably, these rewards can be categorized into two primary sources: those originating from the *consensus layer* (Beacon chain) and those from the *execution layer*. Within the Beacon chain, rewards are issued to validators in recognition of their pivotal role in the consensus mechanism. Consensus is achieved through the integration of a fork choice rule, LMD-GHOST, and a finalization mechanism, Casper FFG. The LMD-GHOST (Latest Message Driven Greediest Heaviest Observed SubTree) protocol directs validators to extend the canonical chain by ensuring that the block with the highest attestation weight is selected as the head of the blockchain. Casper FFG (Friendly Finality Gadget) complements this by securing safety through finalizing blocks based on a two-thirds supermajority vote^23^. The correct execution of these mechanisms is incentivized by the protocol. In contrast, the execution layer introduces two distinct types of rewards:

1. **Gas Fees Accumulation**: This component involves the accumulation of *gas fees*, which users pay to facilitate the inclusion of their transactions within a block^24^.
2. **Maximum Extractable Value (MEV) Extraction**: MEV is associated with the value that validators can extract from the ordering and inclusion of transactions within newly created blocks^25^. Validators have the option to optimize this process by outsourcing such responsibilities to block builders, and specialized agents within the ecosystem, such as *Flashbots*^26^.

Our research focus revolves around a comprehensive examination of the reward mechanisms inherent to the Ethereum Beacon chain. Our primary objective is to illuminate its role in nurturing active participation and ensuring the long-term stability of the Ethereum network. It is of utmost importance to underscore that the rewards disbursed at the Beacon chain layer significantly contribute to the expansion of the monetary supply, denominated in Ether, within the system.

Validators, entities holding stakes of 32 Ether within the Beacon chain deposit contract, receive rewards for engaging in three distinct roles within the Proof-of-Stake consensus process: *attestations*, *block proposers*, and members of the *sync committee*:

1. **Attestors** are entitled to rewards for attesting to the following:
  - *Source*: Voting in favor of a source checkpoint for Casper FFG.
  - *Target*: Voting in favor of a target checkpoint for Casper FFG.
  - *Head*: Voting for a chain head block for LMD-GHOST.
2. **Block Proposers** receive rewards in three different categories:
  - *Attestation*: Inclusion of attestations in a Beacon chain block.
  - *Sync Committee*: Incorporation of the sync committee’s output.
  - *Whistleblowing*: Reporting instances of malicious behavior, which encompasses:
    i. *Proposer Slashing*: Reporting a slashable violation by a proposer.
    ii. *Attestation Slashing*: Reporting a slashable violation by an attestation.
3. **Sync Committee Members** play a pivotal role in assisting light clients in maintaining a synchronized record of Beacon block headers.

**Fig. 2:**
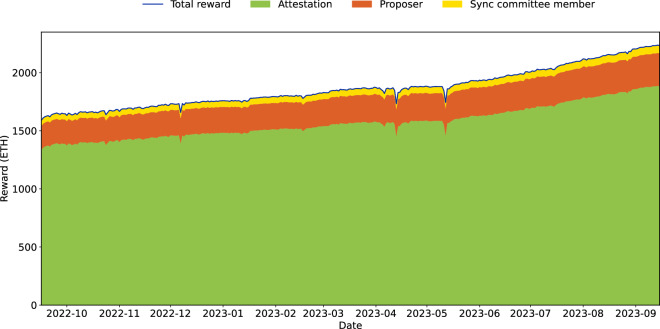
Daily attestation, proposer, and sync committee reward.

**Fig. 3:**
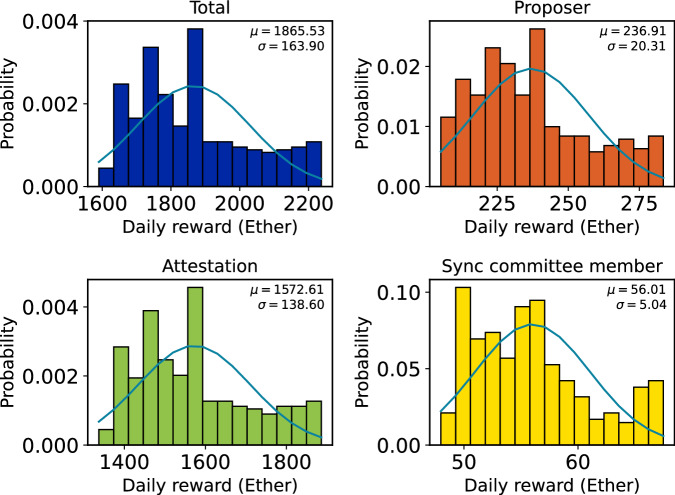
Distributions of the daily total, attestation, proposer, and sync committee rewards.

**Fig. 4:**
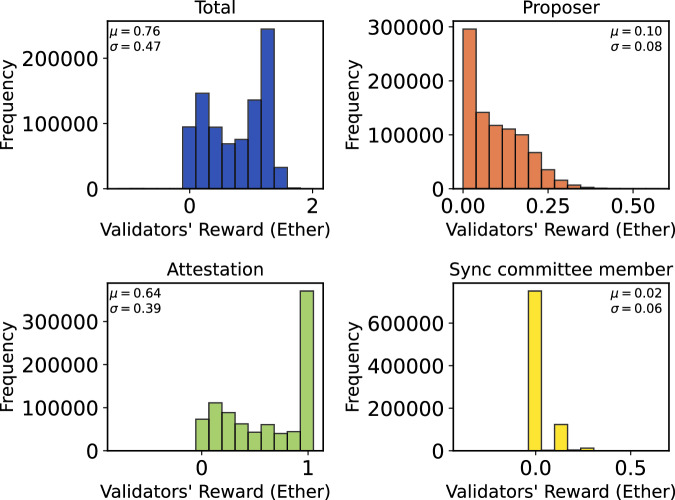
Distributions of the total, attestation, proposer, and sync committee rewards among validators.

The categories are in descendant order of occurrence. Validators, while they are active, will have a slot in which they are asked to provide their votes (see above) for each epoch. The proposer duties can only occur 32 (number of slots) times in an epoch since validators are chosen randomly, each validator will face this duty less often. Finally, Sync Committees are assigned only every 256 epochs and are formed of 512 validators. This categorization establishes the groundwork for an in-depth analysis of the PoS Ethereum’s reward distribution mechanisms within its Beacon chain consensus layer. Figure 2 illustrates a time series of the rewards from September 15, 2022 to September 15, 2023. The Figure shows the daily aggregated rewards on the Beacon chain, categorized into three distinct types: proposer reward, attestation reward and sync committee member reward. The blue line represents the total daily reward which is the sum of these three rewards issued on the Beacon chain. Figure 3 shows histograms of the (daily) aggregated rewards, also divided into the total reward and the other three categories. On average, 1865.53 Ether are generated daily on the Beacon chain. Out of this total, 236.91 Ether are assigned to proposers, 1572.61 Ether are allocated to attestations, and 56.01 Ether are allocated to Sync committee members. Figure 4 shows the histograms of the income of validators (aggregated over the entire study period). On average, a validator can earn 0.76 Ether from September 15, 2022, to September 15, 2023, and can consistently earn around 0.64 Ether by attesting blocks during this timeframe.

### Application of decentralization metrics

**Fig. 5:**
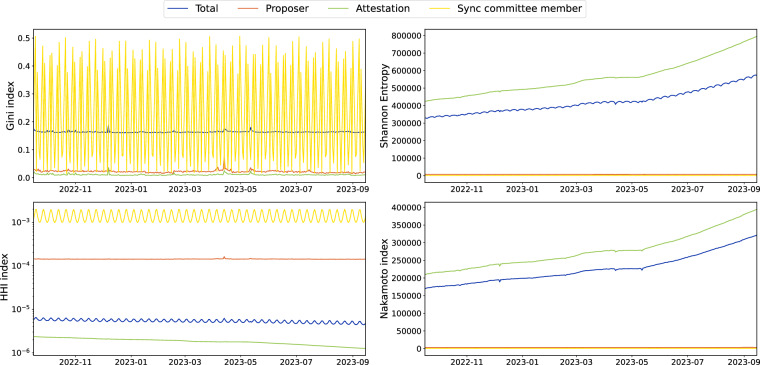
Decentralization metrics: The Gini index (top left), Shannon Entropy (top right), HHI (bottom left) and the Nakamoto index (bottom right) are accumulated and split into the single reward-bearing categories. All these metrics measure the reward decentralization among validators who have received corresponding rewards.

Asahi *et al*.^9^ conducted an investigation into the distribution of wealth in eight major cryptocurrencies, including Bitcoin and the Proof-of-Work (PoW) Ethereum. Their findings revealed that, despite the purported emphasis on decentralization within various blockchain networks, wealth distribution remained unequal, with the notable exception of Dash coin. To assess the decentralization of the PoS Ethereum, we employ several decentralization metrics as outlined in “SoK: Blockchain Decentralization”^12^, including the Shannon entropy, Gini Coefficient, Herfindahl-Hirschman Index (HHI) and Nakamoto Coefficient. These metrics offer valuable insights into the concentration of rewards among different stakeholders. Figure 5 illustrates the temporal variations in reward distribution across different reward types (total, proposer, attestation and sync committee member) using four inequality and decentralization metrics: Gini Index, HHI Index, Shannon Entropy and Nakamoto Index.

## Data Records

### Data summary

The dataset comprises three main types of validator rewards on Ethereum’s Beacon chain: proposer rewards (collected per slot), attestation rewards (collected per epoch), and sync committee rewards (collected per slot). The data spans from September 15, 2022 to September 15, 2023 and consists of information from 895,203 validators. The attestation reward dataset on an epoch basis is particularly large at 1.6 terabytes in size. By combining epoch numbers with their corresponding timestamps, we aggregated these rewards on a daily basis, resulting in a comprehensive *total_reward_by_date* file (3.3 G in parquet format) that tracks each validator’s daily earnings across all reward types. This daily reward distribution data serves as the foundation for calculating decentralization indices, enabling us to assess the concentration of rewards among validators over time.

The comprehensive dataset detailing final reward records for Ethereum validators is securely stored and publicly accessible on the Harvard Dataverse^27^. This dataset encompasses validator rewards from three distinct sources, presented at various frequencies, along with daily decentralization indices. The total_reward_by_date file is in parquet format, while others are formatted in CSV.

### Slot and epoch timestamp

When querying reward data from the Beacon chain, the slot number or epoch number is returned without corresponding timestamps. To gain a more comprehensive understanding of the dynamics of rewards, it is imperative to synchronize the timestamps with each slot and epoch. Fortunately, the time interval between slots and epochs on the Beacon chain is fixed. The smallest time unit on the Beacon chain is a slot, with each slot lasting 12 seconds. Every 32 consecutive slots form an epoch, which lasts for 6.4 minutes. Given that the first slot on the Beacon chain started at timestamp 1606824023(corresponding to December 1, 2020), the time of each subsequent slot can be calculated accordingly. As for the time of each epoch, we take the time of the first slot within each epoch as the epoch’s time. The data structure of slot and epoch timestamp is shown in Table 2.

**Table 2:** Metadata of the slot, epoch, and timestamp data.

Variable	Data Type	Unit
slot	int64	count
epoch	int64	count
timestamp	int64	count

### Proposer, attestation and sync committee reward

The proposer reward data is collected from the Teku node on a per-slot basis. This data consists of various fields, as shown in Table 3. The total_proposer_reward field represents the sum of other reward types, while the epoch is generated based on the slot number. Similarly, the sync committee reward is obtained on a per-slot basis and comprises the fields: epoch, slot, validator_index and reward. The attestation reward can only be acquired per epoch and includes the fields: epoch, validator_index, head, target, source and total_attestation_reward. Furthermore, validators may face penalties, resulting in negative rewards, if they fail to fulfill their duties such as missing a slot or providing invalid proposals or attestations. The structure of each reward data type is presented in Table 3.

**Table 3:** Comprehensive metadata for reward data files.

File Name	Variable	Data Type	Unit
Proposer Reward	epoch	int64	count
	slot	int64	count
	validator_index	int64	count
	total_proposer_reward	int64	Ether
	attestations	int64	Ether
	sync_aggregate	int64	Ether
	proposer_slashings	int64	Ether
	attester_slashings	int64	Ether
Sync Committee Reward	epoch	int64	count
	slot	int64	count
	validator_index	int64	count
	sync_reward	int64	Ether
Attestation Reward	epoch	int64	count
	validator_index	int64	count
	head	int64	Ether
	target	int64	Ether
	source	int64	Ether
	total_attestation_reward	int64	Ether

This table provides an organized overview of metadata for various data files related to the proposer, attestation, and sync committee rewards, detailing variables, data types, and units.

### Total rewards

After obtaining the proposer reward, attestation reward and sync committee reward, we combine these rewards using the epoch number and validator index as key identifiers. The attestation reward dataset is significantly large, totaling 1.6 terabytes in size. To effectively process and analyze this data, we apply the epoch timestamp to each dataset. This allows us to categorize the data on a daily basis and aggregate the rewards accordingly. Finally, we merge the three datasets using the date and validator index. This process results in the creation of the total_reward_by_date file, which is in parquet format and has a size of 3.3 G. This dataset shows different types of rewards received by each validator who participates in the validation process of the consensus on a daily basis. Table 4 presents comprehensive metadata fields for rewards by epoch and rewards by date, offering a detailed overview of the aggregated rewards.

**Table 4:** Comprehensive metadata for total rewards.

File Name	Variable	Data Type	Unit
Total Reward by Epoch	epoch	int64	count
	validator_index	int64	count
	total reward by epoch	int64	Ether
	attestation reward	int64	Ether
	sync committee reward	int64	Ether
	proposer reward	int64	Ether
Total Reward by Date	date	date	count
	validator_index	int64	count
	total reward by date	int64	Ether
	attestation reward	int64	Ether
	sync committee reward	int64	Ether
	proposer reward	int64	Ether

This table provides an organized overview of metadata for total rewards categorized by epoch and date, including details of variables, data types, and units.

## Technical Validation

To verify the accuracy of our dataset, we employ two validation methods. The first method involves cross-checking the total daily rewards issued on the Beacon chain. We refer to the “Total Daily Income (Ether)” chart on Beaconscan at https://beaconscan.com/stat/validatortotaldailyincome. This chart displays the total rewards received by all validators in Ether each day. We compare this data with the daily total rewards shown in Fig. 2 to ensure consistency. Moreover, to ensure accurate validation of rewards assigned to a specific validator for a designated time frame, such as an epoch or a day, the “Income detail history” API method from the beaconcha.in website at https://beaconcha.in/api/v1/docs/index.html is a valuable tool. This method provides a detailed breakdown of the income components earned by the validator during the specified epoch. It is important to note that the total income from this data source includes not only Beacon chain income but also transaction fees from the execution layer and MEV (Maximal Extractable Value) rewards. Initially, this API allows retrieval of reward details for a single validator by inputting the validator’s index number and the epoch number on the website. In order to ensure data accuracy, instead of using the default API settings to validate the correctness of reward data for a validator at a specific epoch, we have adapted this API method into a Python function in our GitHub repository which allows us to retrieve reward data for any validator on any given day from the beaconcha.in website. This will help us verify the accuracy of the *total_reward_by_date* file. Since both daily reward datasets are aggregated from the epoch time scale, if the reward results remain consistent on a daily basis, it indicates that the dataset we have collected and formed is reliable.

Our verification process is as follows: First, we randomly select *n* validators. For each validator, we randomly choose a date between September 15, 2022 and September 15, 2023. Next, we retrieve the reward details for these validators on the selected date from our dataset. Then, we utilize the API calls method to fetch the daily rewards data of these validators from the beaconcha.in website. Finally, we compare the two datasets by merging the obtained datasets into one table. In Table 5, we can observe the alignment between the data from the beaconcha.in API and our examined data.

**Table 5:** Data validation.

date	Data we collected and generated(GWEI)					Data from the beaconcha.in API(GWEI)			
	validator index	total reward	attestation reward	proposer reward	sync committee reward	total reward	attestation reward	proposer reward	sync committee reward
27.10.2022	89175	3147217	3147217	0	0	3147217	3147217	0	0
30.10.2022	49579	3137206	3137206	0	0	3137206	3137206	0	0
01.11.2022	59176	3126813	3126813	0	0	3126813	3126813	0	0
10.11.2022	11029	2467138	2467138	0	0	2467138	2467138	0	0
16.11.2022	65067	3098273	3098273	0	0	3098273	3098273	0	0
13.01.2023	14720	2993459	2993459	0	0	2993459	2993459	0	0
24.01.2023	49827	2992321	2992321	0	0	2992321	2992321	0	0
09.02.2023	97351	2962964	2962964	0	0	2962964	2962964	0	0
28.02.2023	46616	2934895	2934895	0	0	2934895	2934895	0	0
04.03.2023	69692	2913092	2913092	0	0	2913092	2913092	0	0

The table presents the consistency between the data collected and the data from the beaconcha.in API. We randomly selected 10 validators and 10 dates to compare their total reward, attestation reward, proposer reward, and sync committee reward. The results indicate that the data is consistent.

It is worth noting that although the beaconcha.in API provides reliable Beacon chain reward data, calls are limited to 10 requests per minute per IP with the free-tier account. Considering the presence of over 1 million validators on the Beacon chain, it is not feasible to obtain the complete reward dataset as presented in this paper.

## Usage Notes

### Applicability

The reward dataset provided by this study supports a diverse range of analyses and applications across blockchain research. Below, we outline key research directions that this dataset enables:

1. **Temporal and Predictive Analysis of Rewards**: Our dataset allows for detailed time-series analysis of Ethereum’s Proof- of-Stake (PoS) reward distribution, offering insights into temporal patterns, fluctuations, and shifts in decentralization dynamics^28,29^. Additionally, predictive modeling techniques, such as machine learning, can leverage the dataset to forecast validator behavior, future reward distributions, and network trends to inform protocol improvements and governance.
2. **Decentralization Correlations Across Blockchain Layers**: The dataset provides decentralization metrics at the consensus layer, serving as a foundation for exploring correlations between consensus-level decentralization and other layers, such as hardware, data, network, and application layers^12,30–33^. This approach facilitates a holistic understanding of how decentralization metrics interact across the blockchain architecture.
3. **Comparative Studies of PoS and PoW Mechanisms**: By comparing reward distributions across Ethereum’s PoW and PoS stages, researchers can analyze the impact of the Merge on decentralization and reward fairness^34–36^. Previous research has analyzed participant behaviors in Proof-of-Work systems, such as detecting selfish mining behaviors^37^. However, identifying validator misbehaviors in Proof-of-Stake presents new challenges and requires different analytical approaches. Our dataset provides comprehensive validator reward records that can be utilized to develop novel detection methods for abnormal validator behaviors, such as strategic timing of attestations or inconsistent performance in assigned duties.
4. **Machine Learning Applications**: The dataset is well-suited for a variety of advanced machine-learning applications, enabling deeper insights and optimizations in Ethereum’s Proof-of-Stake ecosystem. For instance, anomaly detection techniques can be employed to identify irregular reward patterns that may signal validator misbehavior, collusion, or systemic vulnerabilities, thereby enhancing network security and integrity^38^. Machine-learning-based clustering and behavioral analyses^39^ can group validators based on reward distribution, performance, or compliance, uncovering systemic trends, reward disparities, and emerging centralization risks. These insights can inform data-driven adjustments to reward structures, promoting fairness and decentralization. Furthermore, integrating on-chain reward data with external datasets, such as social media activity^40^, financial market trends^41^, or governance proposals^42,43^, enables comprehensive analyses of cross-domain interactions and their impact on validator behavior and network dynamics. Additionally, sentiment and governance activities^44,45^ can be analyzed to understand their influence on validator participation and decentralization trends, offering actionable insights for refining incentive mechanisms. Finally, leveraging machine- learning models for data-driven optimization of reward incentives^46,47^ allows researchers to simulate and evaluate changes to reward structures, identify strategies to optimize validator engagement, enhance decentralization, and prevent collusion or monopolistic behaviors within the PoS system.

By offering granular, reusable data, this study facilitates cutting-edge research into blockchain decentralization and PoS system dynamics. The dataset serves as a foundational resource for exploring these topics and encourages the development of data-driven optimizations and insights.

### Future research

While our daily indices provide a comprehensive foundation, we anticipate that future research can further enhance and expand upon them. Potential avenues for future work include:

- **Decentralization Analysis at Entity and Staking Pool Levels**: Our current work analyzed decentralization at the validator level, but the reward data could also be examined at both individual entity and staking pool levels^48–51^. Due to the pseudonymity of validators, entities with significant ETH holdings may split their stakes across multiple validators, potentially obscuring the level of centralization across different scales. Future work could incorporate clustering techniques to link validators to their controlling entities, enabling a deeper understanding of reward dynamics and distribution differences across these levels.
- **Connecting Beacon Rewards to Block Building Rewards**: As mentioned in the Methods section, validators receive transaction fee rewards^52^ for participating in block building, in addition to beacon rewards. Moreover, validators also gain MEV rewards^53^ from the block-building process. Future efforts could incorporate analyses of these additional validator revenue sources.
- **Connecting Blockchain to Real-World Assets**: Our current dataset measures rewards in Ethereum’s native currency. Further research could account for Ethereum’s inflation or deflation over time^54^, as well as exchange rates between Ethereum and other cryptocurrencies or fiat currencies, usually studied in financial technology^55–59^ and blockchain interoperability studies^60,61^. This would connect on-chain decentralization to real-world asset values.

In summary, while our work establishes a robust baseline, there are many exciting opportunities to build on it through additional layers of analysis and connections to external factors. We hope our indices spur further decentralization research that encompasses new dimensions and perspectives.

## Data Availability

The datasets and Python codebase employed for the analysis of Beacon chain rewards in the PoS Ethereum framework are available in a public repository on GitHub at https://github.com/learn-want/ETH2.0-reward. This code, primarily developed in Python and encapsulated within Jupyter Notebook environments, facilitates comprehensive investigations into the dynamics of consensus rewards on the Ethereum blockchain. Academics, blockchain developers, and other stakeholders are encouraged to leverage this open-source resource for advanced studies and explorations in blockchain reward mechanisms.
